# A Conceptual Approach to Reduce the Product Gas Crossover in Alkaline Electrolyzers

**DOI:** 10.3390/membranes15070206

**Published:** 2025-07-12

**Authors:** Diogo Loureiro Martinho, Torsten Berning

**Affiliations:** 1 Green Hydrogen Systems, 6000 Kolding, Denmark; diogoloureiromartinho@gmail.com; 2 AAU Energy, Aalborg University, 9220 Aalborg, Denmark

**Keywords:** alkaline water electrolysis, Zirfon diaphragm, hydrogen crossover, supersaturation, micro-cracks, bubble diameter

## Abstract

The crossover of the product gases hydrogen and oxygen in alkaline electrolyzer operation is a critical factor, severely limiting the operational window in terms of current density and pressure. In prior experiments, it was found that a large degree of oversaturation of the reaction products in the liquid electrolyte phase leads to high amounts of crossover. We are proposing to reduce this amount of oversaturation by introducing micro-cracks in the Zirfon diaphragm. These cracks are meant to induce the formation of hydrogen and oxygen bubbles on the respective sides, and thereby reduce the oversaturation and amount of crossover. In theory, the size of the bubble corresponds to the size of the cracks, and from our computational fluid dynamics simulations, we conclude that the bubbles should be as large as possible to minimize the ohmic resistance in the electrolyte phase. The results suggest that an increase in bubble diameter from 50 microns to 150 microns results in a 10% higher current density at a cell voltage of 2.1 V.

## 1. Introduction

Alkaline electrolyzers are a promising technology for producing “green” hydrogen out of regenerative energy sources like wind and solar. Compared to competing technologies like proton exchange membrane (PEM) electrolyzers and solid oxide (SO) electrolyzers, they are generally considered more mature and have lower CAPEX [1,2], while on the other hand, the efficiencies are generally expected to be lower, resulting in higher electricity consumption and OPEX. Thus, the roll-out of this technology depends strongly on the local electricity price, and countries with lower electricity cost like the USA, Canada, and Norway might favor alkaline technology while other European countries experience higher electricity prices and may favor technologies with higher efficiencies. The manufacturer Cummins, for example, offers PEM electrolyzers with a system-specific electricity consumption of ≤51 kWh/kg hydrogen at a maximum pressure fo 30 bar and alkaline electrolyzers that consume 55–60 kWh/kg hydrogen at a maximum pressure of 10 bar [3]. Thus, the efficiency of an alkaline electrolyzer can be as much as 20% lower compared to PEM technology. If the efficiency of alkaline technology could be further improved, it could become the dominant technology. In an alkaline electrolyzer, the following half-cell reaction takes place at the anode:

(1) $$2{\mathrm{OH}}^{-}\to H_{2}O+\frac{1}{2}O_{2}+2e^{-}$$

At the cathode side, the half-cell reaction is

(2) $$2H_{2}O+2e^{-}\to H_{2}+2{\mathrm{OH}}^{-}$$

Hence, the overall reaction for the electrolysis process is the decomposition of water into hydrogen and oxygen:

(3) $$H_{2}O\to \frac{1}{2}O_{2}+H_{2}$$

**Figure 1:**
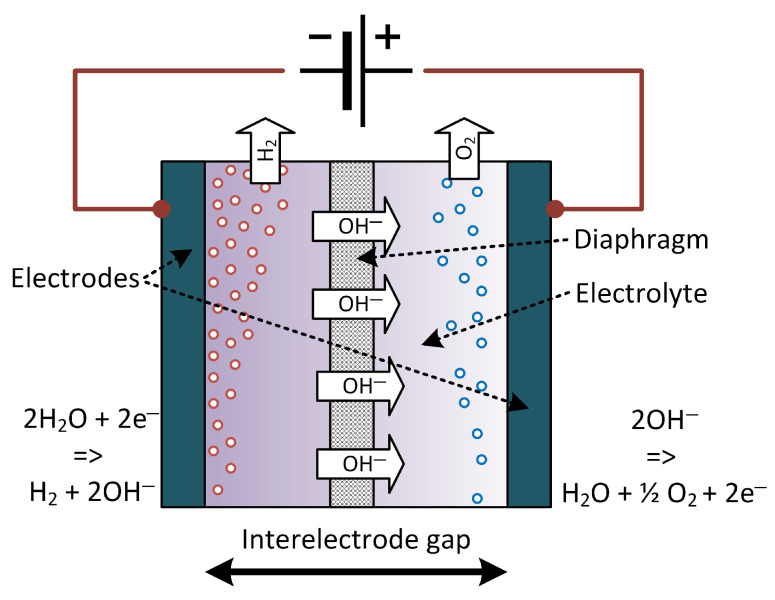
Schematic of an alkaline electrolyzer.

The molar amount of hydrogen is thus twice the molar amount of oxygen produced. In alkaline technology, a mixture of liquid electrolyte and water is circulated to provide the reactants and allow for ionic conductivity. The reaction products are predominantly in the gas phase, but it is known that hydrogen and oxygen also dissolve into the liquid electrolyte phase. Clearly, the gas bubbles provide an obstacle to ionic transport, and especially at high current densities contribute to the ohmic losses; this is why alkaline electrolyzers are usually operated at lower current densities compared to PEM electrolyzers [1]. In a modeling study employing the methods of computational fluid dynamics, it was found that the volume fraction of the gas phase can exceed 50%, thus substantially increasing the ohmic losses [4]. A schematic of an alkaline electrolyzer cell is shown in Figure 1. In conventional designs, there is an interelectrode gap, whereas in zero-gap design, the porous electrodes are in direct contact with the diaphragm to reduce ohmic losses, and the gases emerge behind the electrodes.

In the center of an alkaline electrolyzer is a diaphragm, often Zirfon^TM^ Perl UTP 500, that separates the anode and cathode. Zirfon consists of a porous, mesh-like polyethylene support, while crosslinked polyvinyl alcohol (PVA) may act as a selective skin layer [5]. This separator must allow for the crossing of the hydroxides while preventing the reaction products hydrogen and oxygen from mixing. At 500 microns, this diaphragm is comparatively thick, adding to the ohmic resistance. Zirfon was measured to have an electrical resistance of >0.25 Ω cm^2^ at room temperature, which decreases to <0.15 Ω cm^2^ at 80 °C [6]. A higher porosity leads to lower ohmic resistance, but also to a higher product crossover. Overall, the diaphragm should be thin, hydrophilic, possess a high ion conductivity, and have a high bubble point to prevent gaseous product crossover.

Kim et al. [7] manufactured porous separators with thin selective skin layers to reduce the hydrogen permeation. The thin skin layer was based on crosslinked polyvinyl alcohol (cPVA) and it was fabricated on a porous substrate by a facile and scalable ultrasonic spray coating process. It was observed that the hydrogen permeability was reduced with the number of coating layers while the membrane resistance remained nearly constant up to a certain thickness. In the best case, the optimized separator with a cPVA skin layer combined a low ionic resistance of 0.267 Ω cm^2^ at 30 °C, a bubble point pressure of 2.71 bar, and a low hydrogen permeability of 1.12 × 10^−11^ mol ${\mathrm{cm}}^{-2}$ $s^{-1}$ ${\mathrm{bar}}^{-1}$.

In addition to the commonly used Zirfon, other ion exchange membranes or ion solvating membranes have been successfully tested [8] and compared with the Zirfon^TM^ Perl UTP 500 diaphragm as a reference. Besides physical characterization, the material samples were evaluated electrochemically to determine the ohmic resistance and the product gas impurities. The authors conclude that the thinner diaphragm outperforms the reference material and that polymer membranes can compete with the performance of the reference material [8].

Recently, Luo et al. [5] manufactured a thin V-Zirfon-350 μm separator with a porous, crosslinked polyvinyl alcolhol (PVA) skin, which is attractive as skin layer owing to its hydrophilicity, water permeability, good mechanical properties, and film-forming ability [5]. These authors realized a very low areal resistance (<0.17 Ω cm^2^) and an “ultra-tiny” amount of electrolyte permeation. However, in order to retain electrolyte permeability, a pore-forming agent such as polyvinyl pyrrolidone (PVP) had to be used. The resulting structure suggested micro-cracks inside the skin layer. It is the role of the size of such micro-cracks that is the subject of this communication.

## 2. Crossover in Alkaline Electrolyzers

The overall efficiency of an alkaline electrolyzer is the product of the Faradaic efficiency and the electrical efficiency [9]. The electrical efficiency accounts for the energy consumption relative to the theoretical minimum energy required for water splitting, and it includes losses such as the activation overpotential and ohmic overpotential, while the Faradaic efficiency measures how effectively charge passed through the system contributes to the desired electrochemical reactions [9].

With respect to hydrogen, the Faradaic efficiency is given by [10]

(4) $$\eta_{H_{2}}=1-\frac{N_{H_{2}}^{cross}+N_{H2}^{recomb}+\frac{I_{short}}{2F}}{\frac{I}{2F}}$$

where I/2F is the molar amount of product gas produced out of the current *I*, $I_{short}$ is caused by electrical currents through the membrane, $N_{H_{2}}^{recomb}$ is the loss of hydrogen owing to undesired side reactions, and $N_{H_{2}}^{cross}$ is the loss of hydrogen due to crossover from the cathode side to the anode side, which is the main subject of this work. Generally, the Faradaic efficiency typically ranges from 90% to even higher values under standard operating conditions [11]. The bigger problem of the gas crossover is that a potentially explosive mixture might be formed if too much hydrogen is mixed with the anode oxygen. Therefore, a fundamental understanding of the crossover mechanisms is imperative.

Trinke et al. [12,13] found that the amount of hydrogen crossover increases almost linearly with current density, and at a current density of 1 A/cm^2^, it amounts to 3–5 mA/cm^2^, depending on the temperature. It also clearly increases with temperature. These authors found that the measured amount of crossover is substantially larger than could be expected when assuming thermodynamic equilibrium between the phases. They distinguished between the different crossover mechanisms, also including the effect of supersaturation, and it was shown that the level of supersaturation increases with the current density. These authors then measured the amount of hydrogen in oxygen in an operating PEM electrolyzer and an alkaline electrolyzer at different operating pressures, and found that PEM suffers from larger crossover. In both cases, the percentage of hydrogen in oxygen decreases with current density. The conclusion of the experimental work by Trinke et al. was that the high amount of crossover in an alkaline electrolyzer cell can only be explained when considering that the liquid phase is supersaturated.

Very recently, Barros et al. [14] quantified the amount of crossover for a zero-gap and nonzero-gap configuration, and they found that in the case of the zero-gap configuration, the crossover corresponds to an oversaturation at the diaphragm–electrode interface of up to 80. The experiments were conducted at room temperature with 12% KOH concentration, which is substantially lower than the commonly used 30% KOH concentration. These authors measured the amount of hydrogen crossover and assumed that all crossover is in the supersaturated liquid phase. In this way, they were able to quantify the level of supersaturation ψ. Generally, the amount of supersaturation in the alkaline electrolyzer was measured to be in the range of 20 < ψ < 60 in the case of a zero-gap design, whereas it was only around 2–4 when an interelectrode gap was included. Clearly, in order to reduce the amount of hydrogen crossover, this supersaturation must be reduced.

Over recent years, our research group has developed a computational fluid dynamics model of an alkaline electrolyzer cell. The model is implemented in Ansys Fluent, version R2021, and it employs the so-called Eulerian approach with a full set of transport equations solved for the liquid phase and for the gas phase, treating the electrodes as porous media. The details of the model are described in reference [4,15]. In order to validate the model, crossover values were used as it is known that very different polarization curves can be obtained with the same cell, and therefore polarization curves are not well suited for model validation, yet [16].

In the model, the underlying equation for the mass transfer between the liquid phase and the gaseous phase that mostly contained the product hydrogen and oxygen was

(5) $${\dot{m}}_{lg}=k_{mi}\cdot \phi \left(c_{eq,i}\psi -c\right)$$

where ${\dot{m}}_{lg}$ is the mass transfer rate between the liquid and gas phase, calculated out of the Sherwood number, and ϕ is the surface area, which depends on the assumed bubble diameter according to

(6) $$\phi =6\cdot \frac{\alpha }{d_{b}}$$

where α is the volume fraction of the gas phase, calculated by the solver in every iteration, and $d_{b}$ is the assumed bubble size. $c_{eq,i}$ denotes the equilibrium concentration of the gas species, as can be calculated via Henry’s law. Finally, ψ is the level of oversaturation. This stems out of the fact that the reaction products according to Equations (1) and (2) occur first in the gas phase, and it takes a certain level of oversaturation for bubbles to occur.

**Figure 2:**
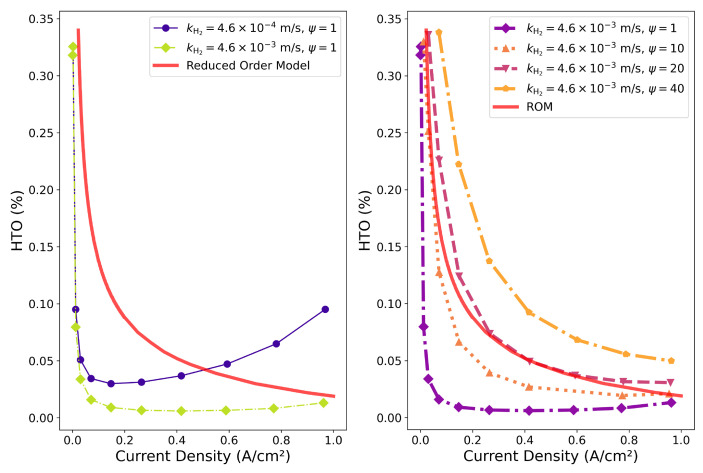
Comparison between experimental (ROM) and calculated hydrogen to oxygen ratio (HTO) at the cathode outlet [15].

Figure 2 shows the results of the hydrogen to oxygen ratio (HTO) of the gas mixture leaving the cathode side as function of the current density that was calculated with our computational fluid dynamics model [4]. The model requires around 20 input parameters, and they are listed in detail in reference [15]. The HTO is strongly dependent on the hydrogen crossover rate. The modeling results are compared with experiments (reduced order model—ROM) for a temperature of 80 °C and the KOH concentration 30 wt%. The experimental results shown are also in very good general agreement with the data published by Trinke et al. [12]. Comparing the modeling results with experiments, it can be seen that the kinetic transfer coefficient $k_{i}$ has an important role in the overall agreement between the model and the experiments. A very good agreement, however, can only be obtained if the supersaturation is increased to a level of ψ=20. This was in accordance with the findings by Barros et al. [14] mentioned above. By reducing this amount of oversaturation, it should be possible to reduce crossover and thus widen the operational window of alkaline electrolyzers in terms of current densities. This brings about the question of whether it is possible to reduce the supersaturation by inducing degassing.

## 3. Reducing the Amount of Crossover

**Figure 3:**
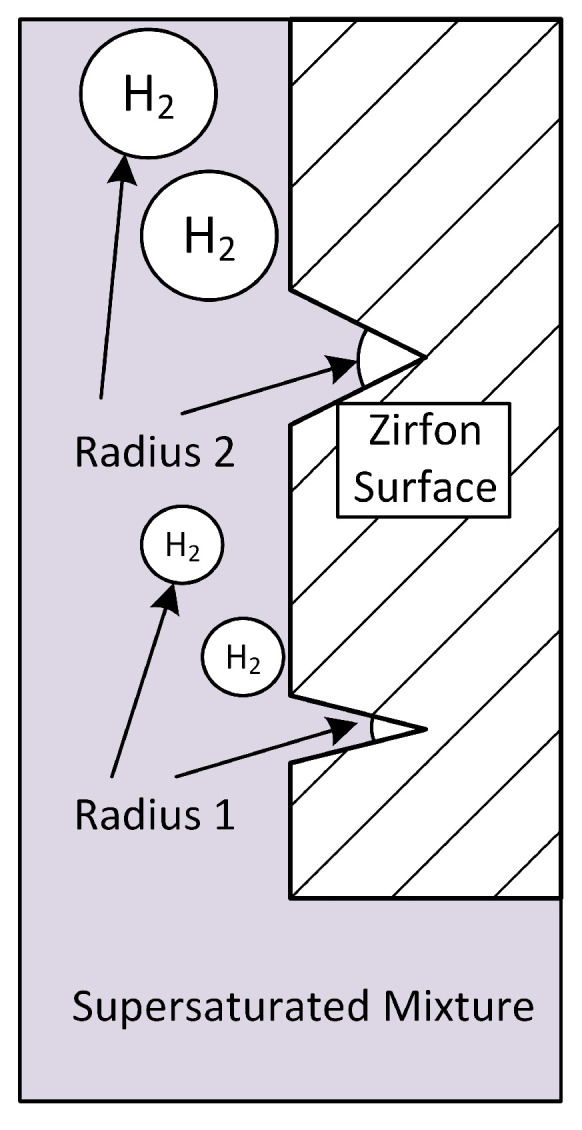
Diaphragm surface with cracks for bubble promotion. Inspired by [17].

Clearly, if the product gases can be made to undergo phase change and produce bubbles, the amount of Fickean diffusion would be reduced. It is a well-known phenomenon that bubble formation can be promoted by introducing cavities in the solid surface area, and reference [17] provides an excellent overview of bubble nucleation in cavities. Even the size of the bubbles can be designed by adjusting the size of the cracks, as suggested in Figure 3. The usual pore size of Zirfon diaphragms was determined to be around 0.15 microns [18]. Therefore, the artificial cracks that we are proposing are more than two orders of magnitude larger than the original pore size of Zirfon.

In the above-mentioned paper by Luo et al. [5], it was found that the introduction of micro-cracks led to a very small electrolyte permeation. Assuming that micro-bubbles were produced by this manufacturing of cracks in the diaphragm which then led to degassing of the liquid phase and lower crossover, the next question concerns the roles that the size of the cracks and resulting bubbles will play.

## 4. Effect of the Bubble Sizes

Our computational model mentioned above was employed to gain a better understanding of the role of bubble sizes in alkaline electrolyzer operation. Bubble sizes of 50 microns and 150 microns were investigated. In multi-phase flow, the size of the bubbles affects the mass transfer coefficient and the drag coefficient between the phases and, along with the gravity effect, has an impact on the calculated volume fraction occupied by the gas phase.

**Figure 4:**
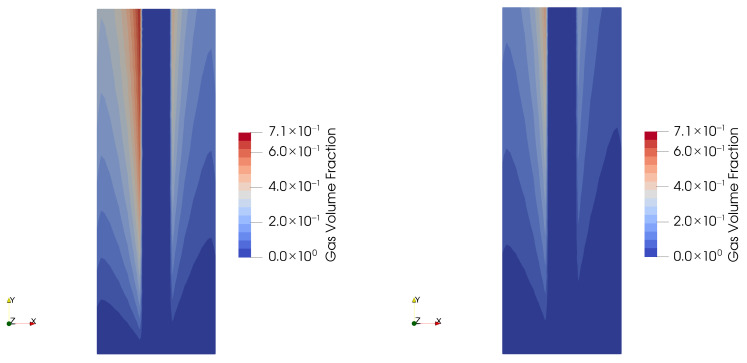
Effect of the assumed bubble size on the predicted gas fraction and the liquid phase velocity for different current densities. (**Left**): 50 microns; (**right**): 150 microns.

Figure 4 shows the calculated gas volume fraction for the two different assumed bubble sizes. In contrast to Figure 1, this is the zero-gap design where the porous electrodes are in direct contact with the diaphragm in the middle, i.e., the interelectrode gap as shown in Figure 1 is equal to the thickness of the diaphragm. However, like in Figure 1, the left side is the anode and the right side is the cathode, separated by the diaphragm, and the general flow direction is from bottom to top. In the case of the smaller bubbles, the predicted gas volume fraction exceeds 71%, close to the diaphragm interface. This causes severe added resistance in the electrolyte phase. In the case of the larger bubbles, the volume fraction occupied by the gas phase is substantially lower, leading to a less reduced electrolyte conductivity.

**Figure 5:**
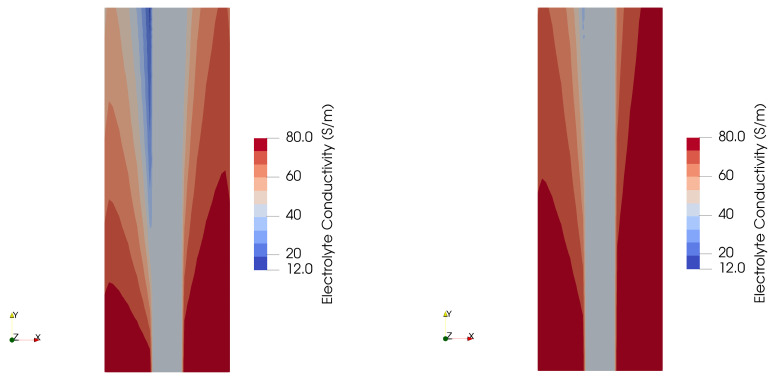
Effect of the assumed bubble size on the predicted electrolyte conductivity. (**Left**): 50 microns; (**Right**): 150 microns.

Figure 5 shows the corresponding calculated electrolyte conductivity for the two different assumed bubble sizes. In the region where the gas volume fraction was predicted to be the highest, the local conductivity is only in the region of 12 S/m. This compares to a value of 80 S/m in the region with only liquid phase, near the inlet in the bottom. In the case of the larger bubble size of 150 microns, the electrolyte conductivity dips to around 50 S/m.

**Figure 6:**
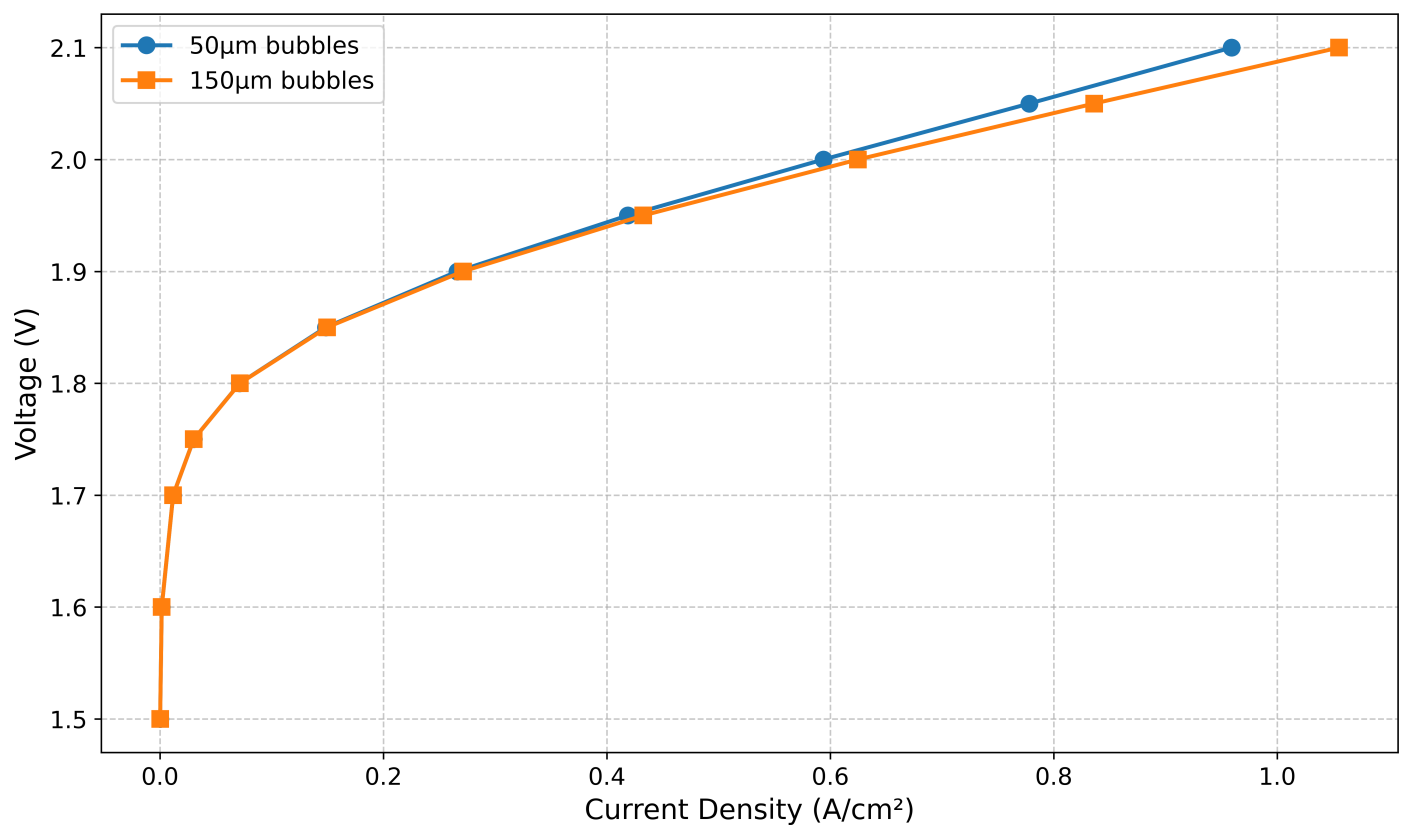
Predicted cell performance for two assumed bubble diameters.

Finally, Figure 6 shows the predicted effect of the bubble diameter on the polarization curve. At a cell voltage of 2.1 V, there is a clear effect of bubble size, with the larger size showing better performance. In detail, the predicted current density for the 50-micron bubbles is 0.96 A/cm^2^, while it is 1.06 A/cm^2^ for the 150-micron bubbles, a difference of 10% in the amount of hydrogen produced. While these simulations give a first indication of the effect of different bubble sizes, important physics such as bubble agglomeration and pressure instability are not yet included in the model, and therefore these results cannot be called conclusive.

## 5. Conclusions

- In order to increase the operating current density in alkaline electrolyzer cells, the Zirfon-like diaphragm has to be carefully optimized, taking into account parameters like the thickness, porosity, permeability, and the bubble point. A high porosity and ensuing permeability of the hydroxides lead to a low ohmic resistance, but also to a higher amount of hydrogen and oxygen crossover, which severely limits the operational window of such electrolyzers.
- The underling reason for the high amounts of hydrogen and oxygen crossover is the oversaturation of the liquid electrolyte phase with these species, leading to high Fickean diffusion. Conceptually, hydrogen and oxygen gas bubbles can be created at the Zirfon surface by manufacturing micro-cracks in it. Ideally, the size of the bubbles created corresponds to the size of the cracks. Typical bubble sizes in alkaline electrolyzers are in the range of 50 microns–200 microns.
- From computational fluid dynamics, we can conclude that larger bubbles lead to a lower ohmic resistance inside the electrolyte phase, and therefore are preferred. The predicted current density at a cell voltage of 2.1 V is 1.06 A/cm^2^ for 150-micron bubbles versus 0.96 A/cm^2^ for 50-micron bubbles.

## Data Availability

Dataset available on request from the authors.
